# Resentment and forgiveness with victims of forced displacement in three cities of colombia

**DOI:** 10.1192/j.eurpsy.2021.1050

**Published:** 2021-08-13

**Authors:** E.P. Ruiz Gonzalez, M.J. Arcos Guzman, M.N. Muñoz Argel, A. Uribe Urzola, A.M. Romero Otalvaro

**Affiliations:** Psychology, Universidad Pontificia Bolivariana, Monteria, Colombia

**Keywords:** armed conflict., Resentment, forgiveness, forced displacement

## Abstract

**Introduction:**

The armed conflict in Colombia manifests and lasts as barbarism in the contemporary world (Zuleta, 2006). Against this background, it is possible to identify among the victims the prevalence of pathologies associated with traumatic events such as forced displacement (Andrade, 2008). Studies indicate a harmony between resentment and other psychosocial effects (Arcos, Muñoz, Uribe, Villamil, Ramos, 2018).

**Objectives:**

The results of the study are presented, which has aimed to analyze the relationship between resentment and forgiveness with victims of forced displacement in three cities of Colombian.

**Methods:**

A correlational study has been carried out with a sample of 40 (n = 40) subjects of which 52.5% are men and 47.5% women, the mean age is 57.52 (σ = 13.591), all with a history of forced displacement; to the data collection has been used the CAPER instrument of Rosales, Rivera and Garcia (2017) (α = .592).

**Results:**

There is evidence of a positive bilateral correlation between the variables studied (r = .000; p = .681), the greater the personal restoration, the greater the feeling of guilt.

**Figure:**
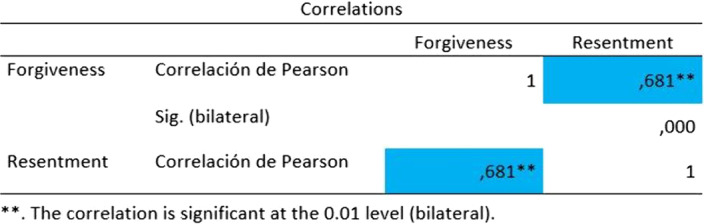

**Conclusions:**

It is important that the intervention processes designed for the victims of forced displacement focused on forgiveness include in their content elements associated with resentment.

